# Development of a simple procedure for the treatment of femoral head osteonecrosis with intra-osseous injection of bone marrow mesenchymal stromal cells: study of their biodistribution in the early time points after injection

**DOI:** 10.1186/s13287-015-0036-y

**Published:** 2015-04-13

**Authors:** Angélique Lebouvier, Alexandre Poignard, Madeleine Cavet, Jérôme Amiaud, Julie Leotot, Philippe Hernigou, Alain Rahmouni, Philippe Bierling, Pierre Layrolle, Hélène Rouard, Nathalie Chevallier

**Affiliations:** Université Paris-Est, Faculté de médecine, Laboratoire de “Bioingénierie cellulaire, tissulaire et sanguine”, EA3952, 5 rue Gustave Eiffel, 94000 Créteil, France; Etablissement Français du Sang d’Ile-de-France, Unité d’Ingénierie et de Thérapie Cellulaire, 5 rue Gustave Eiffel, 94017 Créteil cedex, France; Inserm UMR955, IMRB, 51 Avenue du Maréchal de Lattre de Tassigny, 94000 Créteil, France; Service de chirurgie orthopédique et traumatologie, AP-HP Hôpital Henri-Mondor, 51 Avenue du Maréchal de Lattre de Tassigny, 94000 Créteil, France; Service de radiologie Albert Chenevier, AP-HP Hôpital Henri-Mondor, 51 Avenue du Maréchal de Lattre de Tassigny, 94000 Créteil, France; Inserm U957, Laboratory for Pathophysiology of Bone Resorption, Faculty of Medicine, University of Nantes, 1 rue Gaston Veil, 44035 Nantes cedex 1, France; AP-HP Hôpital Henri-Mondor – A. Chenevier, Service hospitalier, 51 Avenue du Maréchal de Lattre de Tassigny, 94000 Créteil, France

## Abstract

**Introduction:**

Osteonecrosis of the femoral head (ONFH) is a degenerative disease progressing to a femoral head (FH) collapse. Injection of osteoprogenitor cells like bone marrow mesenchymal stromal cells (BMSCs) into the FH appears to be a good therapeutic treatment. However, safety and efficacy of BMSCs to treat bone defect are the main preclinical data required for clinical application. Efficacy and the lack of risk of cell transformation after amplification of BMSCs have been extensively described. The main objectives of this study were to develop a simple and usable procedure for clinicians and control its feasibility by evaluating the biodistribution of BMSCs after injection into the FH in a large animal model. The impact of this approach was evaluated on one natural pig ONFH.

**Methods:**

BMSCs were directly injected in the pig FH, and then the biodistribution of grafted cells was detected by quantitative real-time polymerase chain reaction, cytometry, or a combination of classic histology analysis and *in situ* hybridization (ISH). BMSC efficacy on bone regeneration was evaluated by magnetic resonance imaging (MRI) and histology.

**Results:**

After 30-minute and 24-hour follow-up, grafted cells were detected at the injection site and no BMSCs were detected in filter organs or body fluids. The combination of classic histology analysis and ISH showed a good homogeneity of cell distribution in FH. Local delivery of BMSCs onto a bone scaffold associated with bone formation *in vivo* confirmed the preferential tropism of BMSCs to the bone tissue as well as their efficacy to form bone. Treatment of a natural pig ONFH by autologous BMSCs indicated a beginning of bone healing as early as 2 weeks with a complete healing after 9 weeks. At this stage, MRI and histological analysis were similar to those of a normal FH.

**Conclusions:**

Intra-osseous injection of BMSCs in FH seems to be a good strategy for ONFH treatment as the safety concerning the biodistribution of BMSCs is ensured. Moreover, the efficacy of BMSCs in natural ONFH seems to indicate that this is a promising approach. Altogether, these results constitute the preclinical data necessary for the setup of a clinical application with expanded BMSCs in the context of advanced therapy medicinal products.

## Introduction

Osteonecrosis of femoral head (ONFH) is a progressive degenerative disease due mainly to the loss or compromise of blood flow to the femoral head (FH) and bone progenitor deficiency. If the necrotic bone lesion is not treated early, it may progress to a collapse of FH and require a total hip replacement [1]. This painful disorder commonly occurs in a young population (mean age of 36 years) [2]. To avoid arthroplasty, many conservative procedures are used in the early pre-collapse stage of ONFH, including core decompression associated (or not) with autologous bone marrow (BM) grafting [3,4]. However, even if positive results are obtained, the treatment of the ONFH continues to be a challenging problem for orthopedic surgeons. Bone tissue engineering, using mesenchymal stromal cells (MSCs), provides a promising approach [5]. Indeed, MSCs used in various animal models of bone repair were described to have significant osteogenic potential [6-8], and promising case reports have been published [9,10].

MSCs have the potential to migrate and the capacity to be mobilized to sites of injury. However, it has been shown that injected MSCs via intra-artery and intravenous (IV) portals lead to their detection in the lungs within 15 minutes and then in the liver, kidneys, and spleen, indicating a large spectre of cell dissemination [11-14]. Several articles seem to indicate that if the cells are injected in the site of injury, they stay preferentially and with a better viability to this site compared with an IV injection [13-15]. As bone is the physiological environment of BM-MSCs (BMSCs), we hypothesized that a local delivery of BMSCs into the FH during surgery would facilitate their location and participation in tissue regeneration.

For clinical applications of this advanced therapy medicinal product (ATMP), preclinical data on BMSC safety concerning their innocuity and their biodistribution after their injection are required [16,17]. It has been previously shown that there is no risk of BMSC transformation after their amplification and graft *in vivo* [18]. Currently, it is necessary to demonstrate the homing pattern of the injected cells to avoid inappropriate differentiation in other organs or the development of cancer cells [18-21]. Cells spreading can be impacted by the route of administration [14]. Therefore, to assess the biodistribution, it is essential to administer the cells by the exact portal that will be used in the clinic. Preclinical recommendations require to perform the test with human BMSCs (hBMSCs) on a large animal model of the disease [17]. The pig is considered a translational model in biomedical research because of anatomical, physiological, and biochemical similarities to humans and has been commonly used to obtain preclinical data [22,23].

The goals of our work were to develop a new therapeutic treatment of ONFH by injecting BMSCs directly into the FH without using any BMSC carrier and to make the orthopedic surgery procedure easy and suitable for clinicians. One essential piece of preclinical data required for this procedure is the analysis of their biodistribution in a large animal model. To this end, human or pig BMSCs were directly injected in the pig FH as is done in the clinic for the injection of the concentrated BM with a trocar of 4 mm in diameter. To check whether grafted BMSCs are confined to the target site and not found in proximal tissues or filter organs such as lungs and kidneys or body fluids (blood and BM), we used different highly sensitive techniques like cell cytometry analysis and an innovative approach using species-specific human primers for quantitative real-time polymerase chain reaction (qPCR). In parallel, to localize the human cells, we conducted classic histology analysis associated with *in situ* hybridization (ISH) of the human Alu sequence. To confirm the preferential tropism of BMSCs to bone, a local delivery of BMSCs onto a bone scaffold in a mouse model was performed *in vivo* and BMSC efficacy was also evaluated on one natural pig ONFH.

## Methods

### Animals

Ethical approval for all animal experimentation was obtained from the local ethics committee (ComEth Afssa/ENVA/UPEC, Maisons-Alfort, France) (#12-036) in accordance with the European Guidelines for Animal Care (Directive 2010/63/EU).

#### Pigs

Five female pigs (hybrid of Landrace and large white pigs) with a weight of 35 to 50 kg and age of 3 to 6 months were used (Lebeau Christian, Gambais, France). Pigs were managed in accordance with the instructions of the ethics committee.

#### Mice

Two severe combined immunodeficiency (SCID) mice (males, 7 weeks old) purchased from Charles River Laboratories (Chatillon, France) were used for the ectopic implantation procedure. The mice were anesthetized with isoflurane (Abbott, Rungis, France) and were euthanized with an overdose of pentobarbital (Centravet, Maisons-Alfort, France).

### Biomaterials

Scaffolds of Tutoplast Process Bone (Tutogen Medical, Metz, France) were derived from human cancellous bone. The Tutoplast process consisted of a delipidization, an osmotic cell destruction treatment, hydrogene peroxide treatment, and washing cycles for removal of the non-collagen proteins followed by a solvant dehydrated step and finally a γ-irradiation procedure. Fragments of 2 to 4 mm were cut manually and were stored at room temperature (RT) under sterile condition. Bone scaffolds of equivalent size, volume, and weight (8.0 ± 1.0 mg) were used in this study to ensure a comparable surface area for *in vivo* analyses.

### Bone marrow mesenchymal stromal cell cultures

Pig BMSCs were isolated from BM (5 to 10 mL) of pig humerus (pBMSCs). Human MSCs were isolated from BM (3 to 5 mL) collected from the iliac crest (hBMSCs) of patients undergoing standard BM transplantation procedures (AP-HP Hôpital Henri Mondor, Créteil, France), after having received their informed consent in accordance with the Declaration of Helsinki. The project was approved by the Ethical Committee of Ile de France (section 4 #DC-2009-1049). pBMSCs and hBMSCs were cultured in alpha-modified Eagle’s medium (αMEM) (PAA, Les Mureaux, France) supplemented with 10% of foetal calf serum (FCS) (Stem Cell Technologies, Grenoble, France) and 0.5% ciprofloxacine (Bayer Pharma, Puteaux, France). The hBMSCs used in this study were positive for CD90, CD105, and CD73 and negative for CD34 and CD45 and were able to differentiate into osteogenic, adipogenic, and chondrogenic lineages (data not shown) as previously described [7,24,25].

### Functional characterization

To characterize pBMSCs, their capacity to differentiate into mesenchymal lineages was assessed. For osteogenic differentiation, at 50% confluence the growth medium was replaced by αMEM-10% FCS supplemented with 50 μM L-ascorbic acid-2-phosphate (AA), 10 mM βGlycerophosphate (βGly), 0.1 μM dexamethasone (Dex) (Sigma, Saint Quentin Fallavier, France), and 100 ng/mL rhBMP_2_ (recombinant human bone morphogenetic protein 2, Inductos; Laboratoire Wyeth Pharmaceuticals, Philadelphia, PA, USA). On day 10, the monolayers were fixed in 70% ethanol (Cooper, Melun, France) for 1 hour at 4°C and stained for 15 minutes with Alizarin Red S (Sigma) at RT.

For adipogenic differentiation, at 80% confluence the medium was replaced by a high-glucose medium (Invitrogen, which is part of Life Technologies, Villebon sur Yvette, France) supplemented with 10% FCS, 0.1 mM Dex, 0.2 mM indomethacin, 0.01 mg/mL insulin, and 0.5 mM IBMX (Sigma). On day 10, the monolayers were fixed by using 4% paraformaldehyde (VWR, Fontenay Sous Bois, France) for 5 minutes at RT and stained for 15 minutes with 0.3% Oil Red O (Sigma)/60% isopropanol (VWR). Chondrogenic differentiation was performed in pellet culture by using Stempro, a Chondrogenesis Differentiation Kit (Life Technologies), as described by the manufacturer. On day 21, pellets were fixed in 4% formaldehyde (Sigma) and embedded in paraffin. Sections (3 μm) were stained with Alcian Blue 8GX (Sigma) as described by the manufacturer and counterstained with hematoxylin (Sigma).

### Surgical procedure in pigs

Pigs were managed in accordance with the instructions of the ethics committee. Access to FH was done in accordance with the previously described protocol by a percutaneous approach of the hip [26]. Pigs received an injection of 140 × 10^6^ autologous pBMSCs (n = 1) or hBMSCs (n = 2) in 7 mL of 5% human serum albumin (Albunorm; Octapharma, Boulogne-Billancourt, France), and one pig served as a negative control. To push all the cells inside the FH and to allow the cells to migrate correctly to the necrotic site, a volume of air (2 to 5 mL) was injected and then the trocar was let 5 minutes before being removed.

Blood was collected before (T0) and after injection with a kinetic from 1 minute to 24 hours. Liver, kidneys, spleen, and lungs were collected at either 30 minutes or 24 hours after injection. BM was collected before (T0) and 24 hours after injection. Injected FH and adjacent tissues (that is, capsule, periarticular muscles, gluteus maximus muscle, and round ligament) were analysed 30 minutes after injection. Non-injected pig served as a negative control. Organs were dissected into several pieces and ground on a cell strainer with a suitable piston and used for cytometry and molecular biology.

### Cell labeling with DiOC_18_

Before injection, pBMSCs (20 × 10^6^ cells/mL) were incubated in 10 μg/mL of DiOC_18_ (3, 3′-dioctadecyloxacarbocyanine perchlorate) solution (Molecular Probes, part of Life Technologies) containing 3% FCS for 20 minutes at 37°C [27]. Finally, pBMSCs were washed with 1X Hanks’ balanced salt solution (PAA) three times to get rid of dye remnant and re-suspended in 5% human serum albumin (Albunorm; Octapharma).

### Flow cytometry

Ground organs and body fluids of pigs that received an hBMSC injection were stained for BMSC marker CD73-APC (Becton, Dickinson and Company, Franklin Lakes, NJ, USA) for 15 minutes. Then the different samples of pigs that received an injection of DiOC_18_^+^ pBMSCs (n = 1) or hBMSCs (n = 2) were examined by using FACSCanto™ II (Becton, Dickinson and Company). The data were analysed by using BD FACS DIVA™ software (Becton, Dickinson and Company). The efficacy of labeling was checked before injection with positive expression of stained cells defined as fluorescence greater than 95% of that of the corresponding control pBMSCs not labeled DiOC_18_ or hBMSCs not labeled CD73.

### DNA purification and quantitative real-time polymerase chain reaction

Two specimens were sacrificed 30 minutes and 24 hours after hBMSC injection. Samples were immediately placed in DNA lysis buffer (Qiagen, Courtaboeuf, France) after collection. Total DNA was isolated by using a QIAmp DNA Mini Kit for blood, BM, organs, and tissue samples and using a QIAmp DNA Investigator Kit for FH samples as described by the manufacturer (Qiagen). FH samples were previously pulverized to a fine powder by using a ceramic ball of 6.35 mm and a Fast Prep System (MP Biomedical, Santa Ana, CA, USA). The human genomic DNA (gDNA) obtained was quantified by using human TaqMan Copy Number Reference Assay, RNase P (Applied Biosystems, part of Life Technologies, Courtaboeuf, France) with a 7500HT Fast Real-Time PCR System (Applied Biosystems). hBMSC standard range was realized with decreasing concentrations of hBMSC gDNA diluted in pig gDNA (5 ng/μL). The straight equation (y = −3.526 × 38.163; R^2^ = 0.9983) of standard curve had permitted us to obtain the number of cells corresponding to detected cycle threshold (Ct).

### *In situ* hybridization of human Alu sequences

After euthanasia, half of FH was removed 30 minutes after hBMSC injection, fixed for 48 hours, and decalcified in 4.13% EDTA solution (pH 7.4) (Sigma). After dehydration, clearing, paraffin-embedding, and cutting steps, ISH was performed on the FH sections as previously described by Redwine and Armstrong [28]. Locked nucleic acid-based probes were ordered from Exiqon, Inc. (Woburn, MA, USA), and the sequence used was /5DigN/TCTCGATCTTCCTGACCTCATGA/3Dig_N/. Sections were deparaffinized, rehydrated, washed, and treated with 3% hydrogen peroxide for 15 minutes. After washing, sections were treated in 0.1 M triethanolamine pH 8.0 and 0.25% acetic acid for 20 minutes at RT and pre-hybridized for 1 hour at 56°C in buffer containing 4X SSC (sodium saline citrate) (VWR), 50% deionized formamide, 1X Denhardt’s solution, 5% Dextrane Sulfate, and 100 μg/mL Salmon Sperm DNA. Hybridization buffer was replaced by fresh buffer containing 70 nM of Alu probe and was denatured for 5 minutes at 95°C. Hybridization was carried out for 2 hours at 56°C in a wet chamber. Slides were washed twice for 5 minutes in 2X SSC and twice for 5 minutes in 0.5X SSC at 56°C each. Signals were detected by using anti-DIG horseradish peroxidase-conjugated Fab fragments (Roche, Boulogne Billancourt, France) and diaminobenzidine (Dako, Carpinteria, CA, USA) as substrate. Sections were counterstained with Gill-2 hematoxylin (Thermo Shandon Ltd., Runcorn, UK). Two negative controls were produced to compare and ensure consistent interpretation: negative controls of pig FH injected with human cells in omitting Alu probe and sections of FH injected with physiological saline only exposed to Alu probe. Slides were observed by using a DMRXA microscope (Leica, Nussloch, Germany).

### Magnetic resonance imaging

Magnetic resonance imaging (MRI) was performed on a 1.5-T MRI device (Siemens Avanto, Erangen, Germany). Pigs were in the supine position, and their hind legs were tied in extension. T1-weighted and T2 with fat saturation (T2 FS)-weighted sequences were obtained in the coronal planes.

### Ectopic implantation procedure in immunodeficient mice

Six subcutaneous dorsal pockets (0.5-cm incisions) were prepared in each of the SCID mice. In each pocket, one scaffold was implanted and 300,000 pBMSCs (P2) were injected onto the scaffold in the pocket. The skin was closed with 5-0 sutures (Ethicon, San Lorenzo, Puerto Rico, USA). Cell-free scaffolds were implanted under similar conditions and served as controls. pBMSCs from porcine BM of three independent pigs were tested in duplicate (n = 6 scaffolds).

### Histology

#### Pig femoral head

Pig FHs were fixed with 4% formaldehyde solution (VWR), decalcified in 6.8% nitric acid (VWR) for 2 weeks, and rinsed abundantly in tap water before embedding in paraffin. Sections (3 μm) were stained with Masson’s Tri-chrome (hematoxylin: nuclear staining; acid fuchsin/xylidine ponceau: cytoplasmic staining; light green SF yellowish: collagen staining; all from VWR). Images were visualized by standard light microscopy and captured by using a UC30 Digital Color Camera and CellSens Entry software (Olympus, Rungis, France).

#### Mice scaffolds

After 7 weeks, scaffolds were excised from mice and immediately fixed in 70% ethanol, decalcified for 3 hours in 6.8% nitric acid (VWR), and rinsed in tap water before embedding in paraffin. Sections (3 to 5 μm) were stained with Masson’s Tri-chrome. Fifteen sections of each sample were analyzed (five at the beginning, five in the middle, and five at the end).

## Results

Having previously described that only 7 mL can be injected in the FH (data not shown) [29] and published that a concentration of 20 × 10^6^ BMSCs/mL is efficient to induce bone formation [7,8,24], we decided for the future clinical protocol to inject 140 × 10^6^ BMSCs in the FH. To access cell diffusion after injection in the pig FH, three different protocols were performed: two with human cell origins (hBMSCs) with a 30-minute or 24-hour follow-up and one with autologous pig cells (pBMSCs) with a 24-hour follow-up. After injection, the cells were monitored by flow cytometry, molecular biology, or histology, at different times and in various organs.

### Biodistribution of human bone marrow mesenchymal stromal cells after intra-osseous injection in pig femoral head

**Figure 1:**
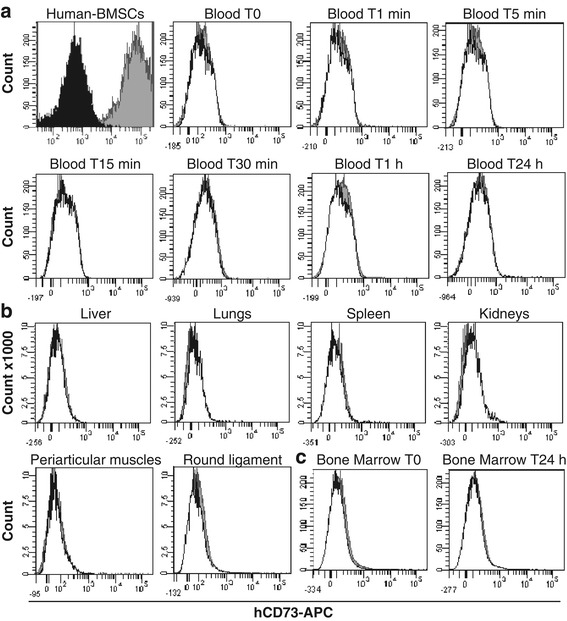
Biodistribution analysis of injected human bone marrow mesenchymal stromal cells (hBMSCs) in pig femoral head by flow cytometry. hBMSCs were injected in the subchondral area of pig femoral head and analysed at different time point. **(a)** Blood was collected before (T0) and after injection with a kinetic of 1 minute to 24 hours. **(b)** Liver, lungs, spleen, and kidneys were analysed either at 30 minutes or 24 hours after injection. Periarticular muscles and round ligament were collected 30 minutes after injection. **(c)** Bone marrow was collected before (T0) and 24 hours after injection. Dark histogram: unstained hBMSCs; grey histogram: stained CD73-APC hBMSCs; and white histogram: negative control cells of a non-injected pig.

Preclinical recommendation required us to assess the biodistribution with human cells [17], and hBMSCs were injected in the pig FH. Compared with unlabeled hBMSCs (dark histogram), the human cells stained for hCD73 were all positive for this marker (grey histogram) (Figure 1). Collected blood at 0 (before injection), 1, 5, 15, and 30 minutes and 1 hour and 24 hours post-injection and collected BM at 0 minutes and 24 hours post-injection were analysed by flow cytometry (Figure 1A, C). Compared with the control blood and control BM (white histogram), no positive cells for hCD73 (grey histogram) were found. Twenty-four hours after injection, no hCD73-positive cells were detected in the liver, lungs, spleen, and kidneys or in adjacent tissues such as periarticular muscles and round ligament (Figure 1B). Only normal tissue-specific cells were found (white histogram).

**Figure 2:**
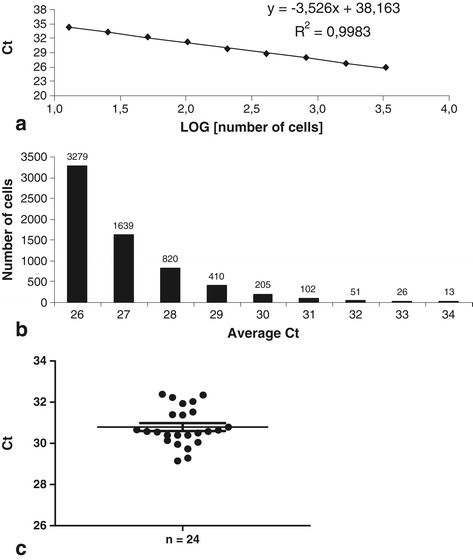
Tracking of injected human cells in pig femoral head by molecular biology. **(a)** Standard curve of human cells quantified by using human TaqMan Copy Number Reference Assay, RNase P. **(b)** Conversion of average cycle threshold (Ct) to corresponding number of cells from the straight equation of the standard curve (y = −3.526 × 38.163). **(c)** Detection (Ct) of injected cells in pig femoral head samples (n = 24) by reverse transcription-quantitative real-time polymerase chain reaction with human RNase P.

To confirm the cytometry study, an approach by species-specific qPCR was developed. To quantify the presence of human cells injected in the porcine FH, a standard curve was established. For this purpose, human gDNA was serially diluted in pig DNA. The linear curve obtained indicated that our qPCR primer (RNase P) was 100% efficient (R^2^ = 0.99) (Figure 2a). This curve allowed us to quantify the exact number of cells up to 13 human cells when mixed in 3,200 pig cells. With this standard curve, we determined our sensitivity threshold at 0.41% (Figure 2B). Below 34 cycles, the standard curve was not linear; human cells were detected but were not quantifiable. Two independent assays were performed: one with a 30-minute follow-up and the other with a 24-hour follow-up. In both cases, no human cells were found in adjacent tissues of FH (n = 6 samples per tissue) or in blood, lungs, liver, spleen, and kidneys (n = 4 samples for blood and n = 6 samples per organ). BM of the non-injected pig humerus was also tested, and as in the other organs, no human cells were found (n = 4) (Table 1). As expected, 30 minutes after human cell injection, the injected hBMSCs were found in the FH. All FH samples tested by RNase P qPCR were positive, with a cycle threshold (Ct) average of 30.8 ± 0.9, which indicated an average of 147 ± 21 cells per 10 mg of FH samples (n = 24) (Figure 2c).

**Table 1:** **Tracking of injected human bone marrow mesenchymal stromal cells** ***in vivo*** **by molecular biology: detection by quantitative real-time polymerase chain reaction of the human RNase P gene**

**Samples**			**Delay after injection**	**RNase detection**
Pig negative control	Organs	Lung	No injection	No detection
		Liver		
		Spleen		
		Kidney		
	Blood			
	Bone marrow			
Pig sample after hBMSCs injection	Adjacent tissues of FH n = 6 samples per tissue	Capsule	30 minutes	No detection
		Periarticular muscles		
		Gluteus maximus muscle		
		Round ligament		
	Organs n = 6 samples per organ	Lung	30 minutes and 24 hours	No detection
		Liver		
		Spleen		
		Kidney		
	Blood n = 4 samples per condition		0	No detection
			1 minute	
			5 minutes	
			15 minutes	
			30 minutes	
			1 hour	
			24 hours	
	Bone marrow n = 4 samples per condition		0	No detection
			24 hours	

FH, femoral head; hBMSCs, human bone marrow mesenchymal stromal cells.

**Figure 3:**
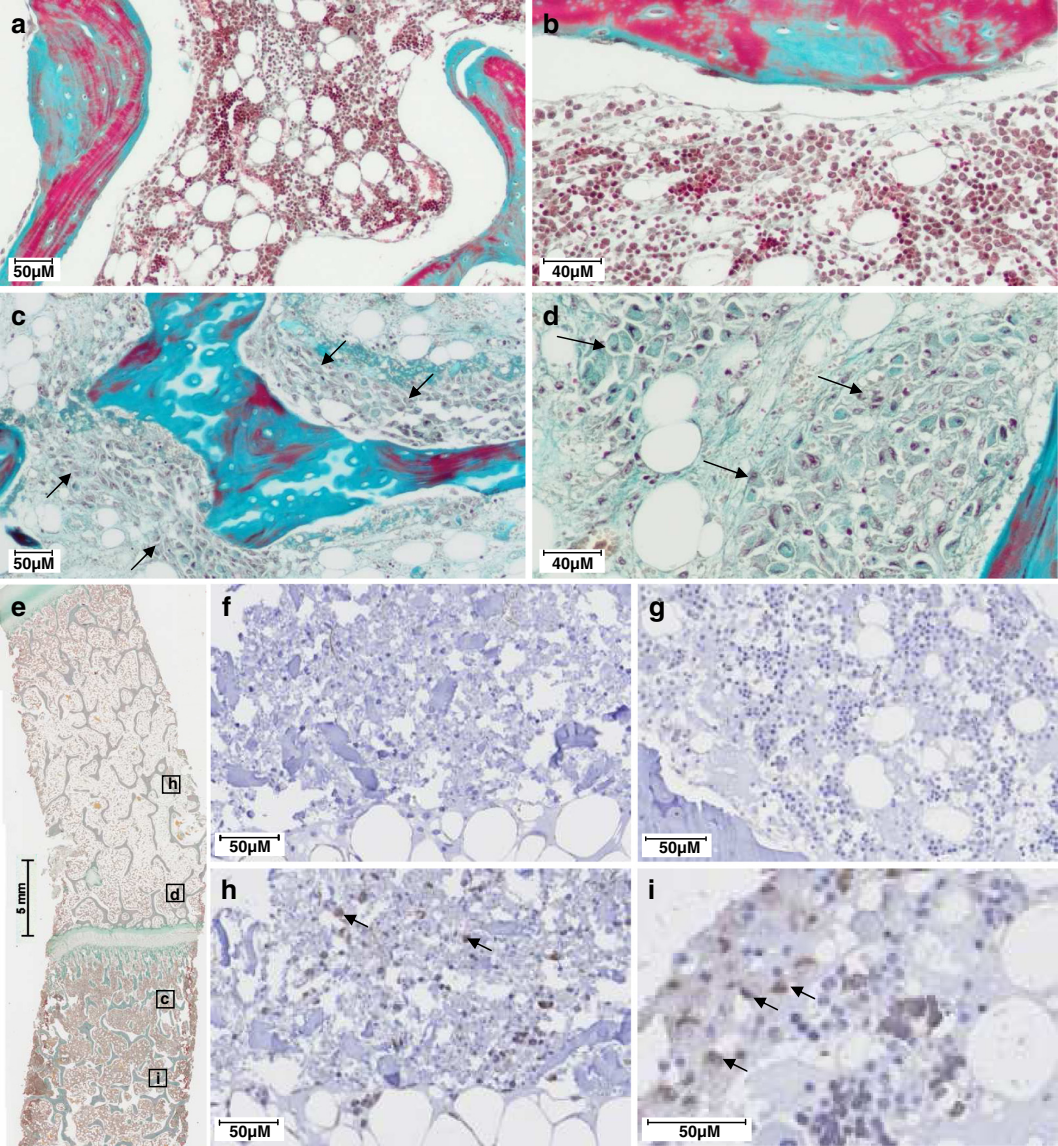
Detection of injected cells in pig femoral head (FH) 30 minutes after human bone marrow mesenchymal stromal cell (hBMSCs) injection. **(a-e)** Masson’s Tri-chrome staining. (a, b) Normal pig FH sections. (c-e) Injected pig FH sections. Arrows indicate the hBMSC area. (e) Localization of sections (c), (d), (h), and (i). **(f-i)** *In situ* hybridization of human Alu sequences (Alu-ISH). (f) Negative control tissue of pig FH injected with human cells in omitting Alu probe stained with the hematoxylin only. (g) Negative control tissue of pig FH injected with physiological saline without human cells, with Alu probe and hematoxylin counterstaining. (h, i) Injected pig FH with human cells detected by Alu-probe staining and counterstained with the hematoxylin. The positive nuclei for Alu-probe staining appear in dark brown (arrows). Magnifications: 10× (a, c, f-h), 20× (b, d), 1× (e), and 40× (i).

Histological analysis was performed to localize the injected cells in the FH. Normal FH showed trabecular bones surrounded by BM endowed with hematopoietic cells and adipocytes (Figure 3a, b). After hBMSCs injection, part of the BM was replaced by large non-hematopoietic cells (arrows). These cells were localized close to trabeculae and seemed to be attached to the bone surface (Figure 3c, d). To confirm histologic findings, a human-specific Alu probe was used for Alu-ISH (Figure 3e-i). Positive brown nuclei staining was considered positive for Alu-ISH. Negative controls did not show any brown cytoplasmic or nuclear staining (Figure 3f, g). The sections of FH with injected human cells showed positive dark brown nucleus staining, whereas other cells were stained only with hematoxylin (Figure 3h, i). Human cells were well distributed inside the pig FH but were localized in the FH subchondral area on either side of the growth plate (Figure 3e). More precisely, dark cells were attached on the bone trabeculae (Figure 3i).

### Biodistribution of autologous pig bone marrow mesenchymal stromal cells after intra-osseous injection in pig femoral head

**Figure 4:**
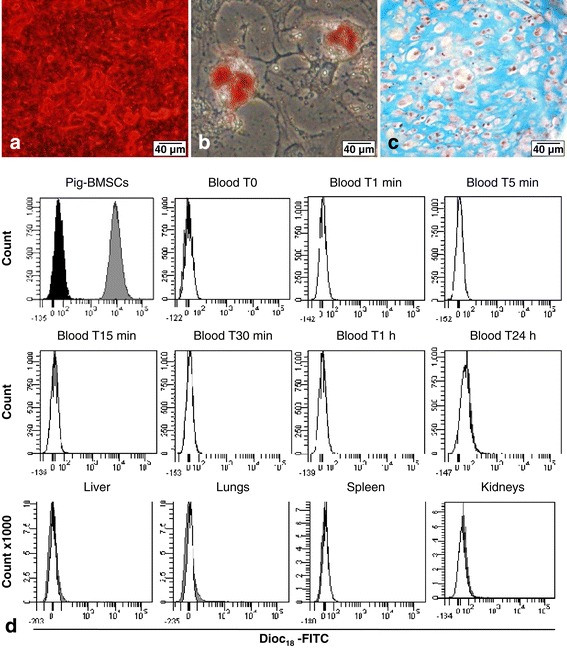
Biodistribution analysis of injected autologous pig bone marrow mesenchymal stromal cells (BMSCs) in pig femoral head by flow cytometry. Characterization of pig BMSCs *in vitro* with **(a)** osteoblastic differentiation with an extensive mineralized matrix of calcium hydroxyapatite crystals stained in red by Alizarin Red S, **(b)** adipocyte differentiation with adipocytes containing lipid droplets in the cytoplasm stained in red by Oil Red O, and **(c)** chondrogenic differentiation with chondrocyte-like cells stained by hematoxylin in purple and surrounded by glycosaminoglycan-rich extracellular matrix stained in blue by Alcian Blue. **(d)** Biodistribution analysis by flow cytometry follow-up during 24 hours (n = 1) of autologous labeled DiOC_18_-FITC BMSCs injected in subchondral area of pig femoral head. Dark histogram: unstained pig BMSCs; grey histogram: stained DiOC_18_-FITC pig BMSCs; and white histogram: negative control cells of a non-injected pig. DiOC_18_, 3, 3′-dioctadecyloxacarbocyanine perchlorate; FITC, fluorescein isothiocyanate.

To confirm our previous results, we wanted to use autologous pig cell grafting. BM from pig humerus was used to isolate pBMSCs and then these cells were characterized by their differentiation properties (Figure 4a-c). Porcine cells deposited an extensive mineralized matrix when cultured for 10 days in the osteogenic medium as demonstrated by strong Alizarin Red staining (Figure 4a). These porcine cells also efficiently differentiated into the adipogenic lineage, as indicated by Oil Red O staining of lipid droplets in the cytoplasm after 10 days of culture in an adipogenic medium (Figure 4b). The chondrogenic potential of pBMSCs was also highlighted by Alcian Blue staining after 3 weeks in a chondrogenic medium. This blue staining revealed a homogenous deposition of proteoglycan within the whole section of the pellet culture, and the counterstaining with hematoxylin showed the presence of chondrocyte-like cells in purple inside the lacunae (Figure 4c).

To be able to follow the autologous injected cells in the pig FH, pBMSCs were stained with DiOC_18_ before injection. Compared with the unstained pBMSCs (control), indicated by dark histogram, the pBMSCs stained by DiOC_18_ (grey histogram) showed a 2 log switch of fluorescence, indicating that 100% of pBMSCs were positive for DiOC_18_ (Figure 4d). No positive cells were found in the different tissues and fluid tested in different times (blood and organs). Only normal tissue-specific cells were found (white histogram).

### Bone tropism of bone marrow mesenchymal stromal cells

**Figure 5:**
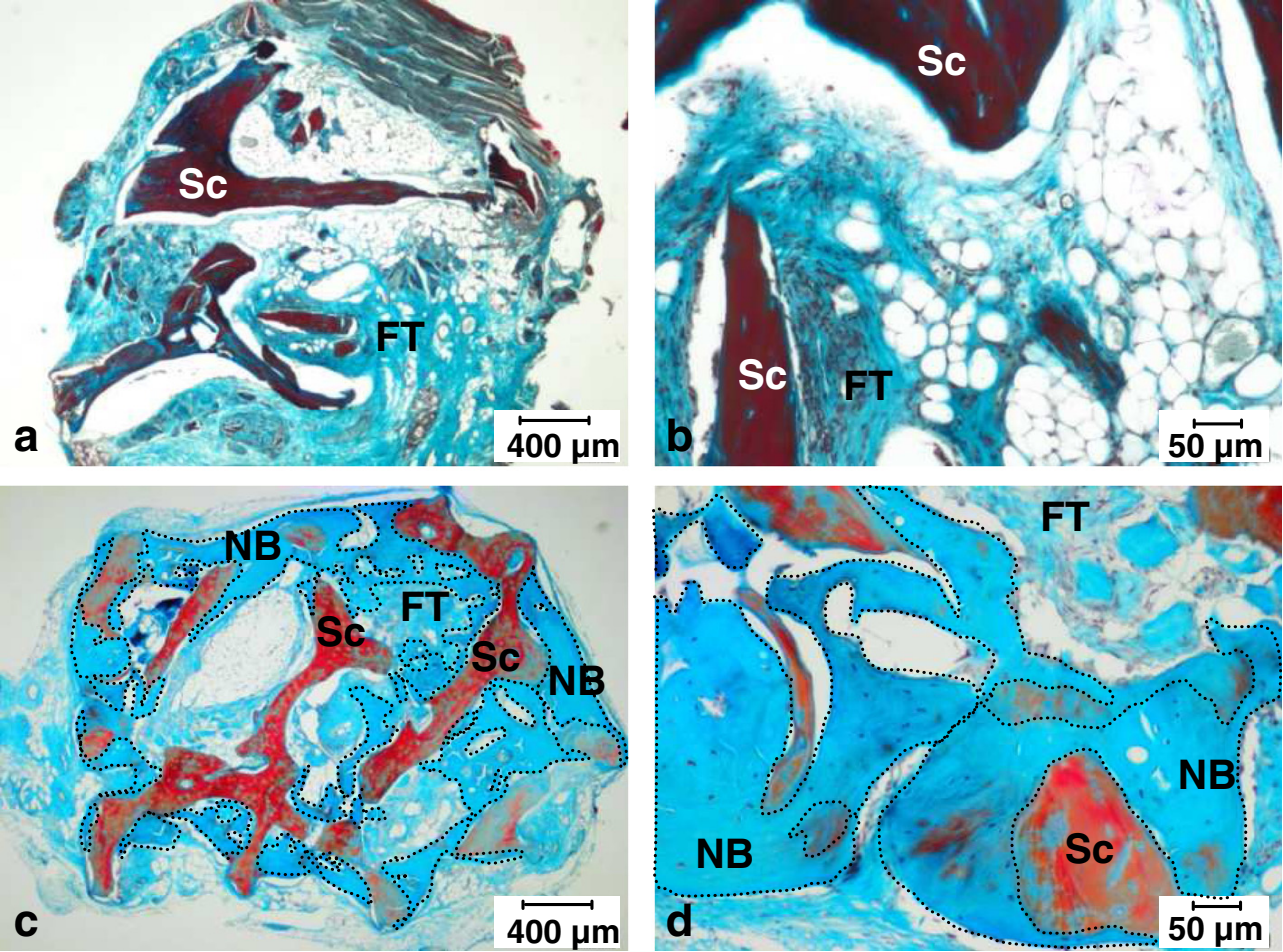
Histological analysis of *in vivo* bone formation after 7 weeks of ectopic implantation into immunodeficient mice. Scaffolds **(a, b)** without pig bone marrow mesenchymal stromal cells (pBMSCs) **(a)** Magnifications: 4×, **(b)** Magnifications: 20×. **(c, d)** with 300,000 pBMSCs directly loaded onto the bone scaffold during the surgery. Cells from three different pigs were tested. **(c)** Magnifications: 4×, **(d)** Magnifications: 20×. Decalcified implants (n = 6 per condition) were embedded in paraffin and stained with Masson’s Tri-chrome (blue/green = collagen and non-mineralized bone; red = mineralized bone scaffold; purple = nuclei; and pink = cytoplasm). Dotted lines correspond to the areas of new bone formation. NB, new bone; Sc, scaffold ; FT, fibrous tissue.

To confirm that BMSCs have a preferential tropism for the bone surface, we assessed the capacity of the cells to locate and induce bone formation after a local delivery of the cells on a bone scaffold. For this purpose, an immunodeficient mouse model of ectopic implantation was performed. In the unseeded controls, no new bone formation was detected, and only scaffold, adipose tissue, and loosely organized connective tissues were present (Figure 5a, b). In contrast, when pBMSCs were added onto the scaffold during the surgery, histological analysis revealed new bone formation with bone tissue containing osteocyte-like cells and osteoblast-like cell lining at the surface (Figure 5c, d). Altogether, this result indicated that pBMSCs preferentially located on bone surface and had the capacity to induce bone formation *in vivo*.

### Cell therapy of natural osteonecrosis of femoral head

**Figure 6:**
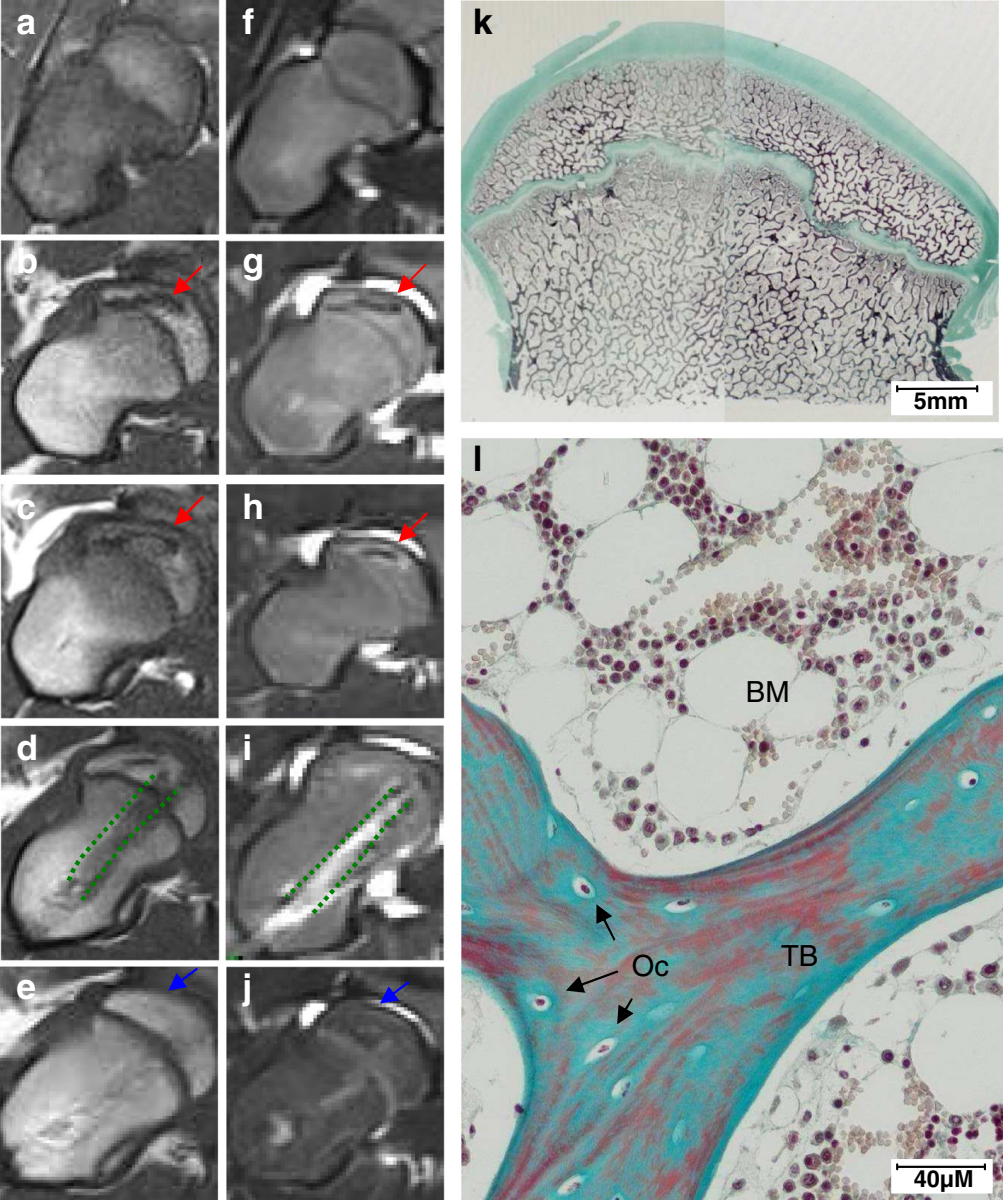
Cell therapy of natural femoral head osteonecrosis (nONFH) in the pig with injection of 140 × 10^6^ autologous pig bone marrow mesenchymal stromal cells (pBMSCs) (n = 1) in femoral head (FH). **(a)** to **(j)** Coronal magnetic resonance imaging (MRI) analysis: red arrows indicate necrotic area, green dashed lines indicate drilling rearrangement, and blue arrows indicate repair area. **(a)** to **(e)** T1-weighted image. **(f)** to **(j)** T2-FS (fat saturation)-weighted image. **(a)** and **(f)** Normal pig FH (negative control). **(b)** and **(g)** Diagnosis of nONFH in pig FH. **(c)** and **(h)** Confirmation of nONFH in pig FH 1 month after MRI diagnosis. **(d)** and **(i)** Pig FH with nONFH 2 weeks after injection of autologous pBMSCs. **(e)** and **(j)** Pig FH with nONFH 9 weeks after injection of autologous pBMSCs. **(k)** and **(l)** Histological analysis of pig FH with nONFH 9 weeks after injection of autologous pBMSCs stained with Masson’s Tri-chrome (blue/green = collagen and non-mineralized bone; red = mineralized bone; purple = nuclei). Magnifications: 1× **(k)** and 20× **(l)**. BM, bone marrow; Oc, osteocytes; TB, trabecular bone.

nONFH was diagnosed in pig. Compared with coronal MRI images of control pig FH (Figure 6a, f), nONFH was observed by a classic MRI appearance of osteonecrosis with a front regeneration: a geographical region of decreased marrow signal within the normally bright fat of the FH on T1-weighted images and mixed low/high signal on T2 FS-weighted images (Figure 6b, g). The front of regeneration delimitated a fatty necrotic area in the epiphysis of high T1 signal and a neutral signal on T2 FS-weighted images. One month after the first MRI, a new MRI analysis was conducted before a possible cell therapy. The diagnosis of osteonecrosis and the absence of spontaneous bone regeneration were confirmed by the observation of results similar to the previous analysis (Figure 6c, h).

After pBMSC injection in the center of nONFH, the pig was able to bear its full weight on the day of surgery. After 2 weeks, the front of regeneration had partially disappeared (Figure 6d, i). Nine weeks after pBMSC treatment, coronal T1 and T2 FS MRI showed no FH collapse and the front of regeneration was almost invisible. MRI FH images presented almost normal fatty bone medullary signal (Figure 6e, j). Then histological analysis was performed. Bone tissue showed a regular trabecular bone network with a normal growth plate in the middle of the FH (Figure 6k). The trabecular bones contained osteocytes and were surrounded by a normal BM constituted with hematopoietic cells and adipocytes (Figure 6l). No features of osteonecrosis such as osteocyte or adipocyte necrosis were visible. Only normal bone was observed.

## Discussion

For ATMPs like hBMSCs, regulatory authorities require safety data concerning their innocuity and their biodistribution after their injection on a large animal model [16,17]. It has been previously shown that there is no risk of hBMSC transformation after their amplification and graft *in vivo* [18]. Although the safety and efficacy of BMSC injection have been tested mainly by using immunodeficient mice, we used the pig as a preclinical model, and for the first time we showed the biodistribution of cell injection by using either autologous pBMSCs or hBMSCs. Our results indicate that BMSCs remained confined at the site of injection and have the potential to repair the osteonecrosis *in vivo*. Currently, there is no single satisfactory method for assessing biodistribution. Several non-invasive, imaging-based monitoring methods have been used to track cell transplants via radiolabels, ferromagnetic particles (that is, iron oxide nanoparticles), and genetically modified cells engineered to express reporter transgenes but these approaches can functionally alter the cells [30-34]. To avoid alteration of BMSC functionality, hBMSCs were directly injected in the FH. Therefore, we used several highly sensitive techniques for cell detection, like flow cytometry, real-time qPCR, histology, and Alu-ISH analysis, in order to validate our results. qPCR is a quantitative and highly sensitive technique which allows the presence of a few human cells to be detected even when they are mixed with pig DNA. Our standard curve indicated that we were able to determine the exact number of human cells up to 13 human cells mixed in 3,200 pig cells. Below, human cells were detected but their number was not quantifiable. In the present study, our data demonstrated that after direct loading, grafted hBMSCs did not have unwanted homing. Indeed, no human cells were detected throughout the kinetic, from 30 minutes to 24 hours after implantation in tissues such as blood, BM, lungs, liver, spleen, and kidneys. Similarly, in tissues located near the drilling injection, such as capsule, round ligament, periarticular muscles, and gluteus maximus muscle, human cells were absent at 30 minutes post-transplant. Human cells remained at the implantation site, in the FH, 30 minutes after cell graft with an average of 147 ± 21 human cells per 10 mg of FH.

The drawback of this technique is the difficulty to clearly discriminate between viable BMSCs or macrophages which contain phagocytized human cell fragments [35]. To this end, qualitative analyses were performed. Because of their high repetition and species specificity, Alu sequences are a marker of choice to use ISH to detect few human cells in pig tissue [16]. This sensitive qualitative technique precisely localizes the cells in FH and indicates a good homogeneity of human cell distribution with the histological analysis. These results confirmed that grafted hBMSCs remained on trabeculae and provided evidence of the absence of spreading of these cells to the other tissues. Even if cells are described as immune-privileged with immunomodulatory, anti-inflammatory, immunosuppression potential and are used for xenogenic transplantation [36,37], we wanted to confirm our results by using autologous cell grafting. For this purpose, pig cells were stained with DiOC_18_, a green fluorescent membrane dye which does not induce adverse cellular effects [38]. The only drawback is that the cells can lose half of their fluorescence after doubling. However, human and pig BMSC doubling time is between 50 and 55 hours [7,39], and our follow-up does not exceed 24 hours. One day after pBMSC injection in the FH, the flow cytometry analysis indicated that the injected cells were detected neither in blood nor in filter organs.

In contrast to studies in which cells are injected intravenously, our results confirm that injected cells preferentially stay in the injected tissue [13,14,40,41]. In our case, this can be explained by the fact that bone is the physiological niche of BMSCs. We confirmed their tropism for the bone surface with the ectopic bone implantation model in immunodeficient mice as only bone scaffold receiving pBMSCs during surgery gives bone formation. Moreover, our results with pBMSCs are in accordance with recent data we published showing that hBMSCs stay on the scaffold and do not migrate to other organs and this was the case throughout the 6 weeks of the study [42]. On the other hand, our data of grafted autologous pBMSCs in nONFH in the pig support that a local delivery of BMSCs into the FH during surgery facilitates their attachment and their participation in tissue regeneration. Altogether, these results indicate that a direct injection of BMSCs on the bone site is a safe procedure, even if a 4-mm diameter trocar is used to inject the cells into the FH. Clearly, these results indicate that there is no need to use a cell carrier like hydrogel or a plug after cell injection to obtain good cell localization without dissemination throughout the body. This is an important point as it was not possible to inject the cells into the FH when cells were mixed with hydrogel (data not shown). Finally, the feasibility of this approach is supported by the commonly used treatment of injection of concentrate BM in FH for the early stages of ONFH. Effectively, no side effects and even a positive effect have been observed in patients [3,4,43].

Our preliminary data for the efficacy of grafted autologous BMSCs in nONFH in the pig seem promising and show a potential of BMSCs in the repair of necrosis *in vivo.* However, nONFH is rare in pigs. To have a statistically relevant result of the effectiveness of BMSCs on this painful disease, it is necessary to test this approach on one of the models of ONFH which has been developed in either emu or pig and to conduct a study on a large number of animals [26,44]. As the effectiveness of BMSCs has been proven and we and others showed the safety of this approach, the second strategy could be to go directly to the clinic [6,7,13,14,18,40,41].

BMSC graft is a promising therapeutic approach for treating ONFH but the other question is the effectiveness of the BMSCs from patients with ONFH. Previous data from Yoo *et al*. described the good osteogenic abilities *in vitro* of BMSCs from patients with osteonecrosis [45]. Altogether, these data indicate that the use of autologous BMSCs for a therapeutic treatment of ONFH is a feasible strategy.

## Conclusions

In summary, we have demonstrated in a large animal model that the intra-osseous portal is a safe and promising strategy for cell therapy treatment of FH osteonecrosis. As it has been previously shown that there is no risk of transformation of hBMSCs after their amplification and graft *in vivo* [18], this new study contributes to the preclinical data which are required for the clinical application of hBMSCs in the context of ATMPs.

## Abbreviations

αMEM: alpha-modified Eagle’s medium
Alu-ISH: *in situ* hybridization of human Alu sequences
ATMP: advanced therapy medicinal product
BM: bone marrow
BMSC: bone marrow mesenchymal stromal cell
Ct: cycle threshold
Dex: dexamethasone
DiOC_18_: 3, 3′-dioctadecyloxacarbocyanine perchlorate
FCS: foetal calf serum
FH: femoral head
FS: fat saturation
gDNA: genomic deoxyribonucleic acid
hBMSC: human bone marrow mesenchymal stromal cell
ISH: *in situ* hybridization
IV: intravenous
MRI: magnetic resonance imaging
MSC: mesenchymal stromal cell
nONFH: natural osteonecrosis of femoral head
ONFH: osteonecrosis of the femoral head
pBMSC: pig bone marrow mesenchymal stromal cell
qPCR: quantitative real-time polymerase chain reaction
RT: room temperature
SCID: severe combined immunodeficiency
SSC: sodium saline citrate
